# Outcome and predictors for successful resuscitation in the emergency room of adult patients in traumatic cardiorespiratory arrest

**DOI:** 10.1186/s13054-016-1463-6

**Published:** 2016-09-06

**Authors:** J. Zwingmann, R. Lefering, M. Feucht, N. P. Südkamp, P. C. Strohm, T. Hammer

**Affiliations:** 1 Department of Orthopedics and Trauma Surgery, Freiburg University Hospital, Hugstetter Str. 55, 79098 Freiburg, Germany; 2 Institute for Research in Operative Medicine (IFOM), University of Witten/Herdecke, Herdecke, Germany; 3 Clinic of Orthopedics and Trauma Surgery, Sozialstiftung Bamberg, Bamberg, Germany

## Abstract

**Background:**

Data of the TraumaRegister DGU® were analyzed to derive survival rates, neurological outcome and prognostic factors of patients who had suffered traumatic cardiac arrest in the early treatment phase.

**Methods:**

The database of the TraumaRegister DGU® from 2002 to 2013 was analyzed. The main focus of this survey was on different time points of performed resuscitation.

Descriptive and multivariate analyses (logistic regression) were performed with the neurological outcome (Glasgow Outcome Scale) and survival rate as the target variable. Patients were classified according to CPR in the prehospital phase and/or in the emergency room (ER). Patients without CA served as a control group. The database does not include patients who required prehospital CPR but did not achieve ROSC.

**Results:**

A total of 3052 patients from a total of 38,499 cases had cardiac arrest during the early post-trauma phase and required CPR in the prehospital phase and/or in the ER. After only prehospital resuscitation (n = 944) survival rate was 31.7 %, and 14.7 % had a good/moderate outcome. If CPR was required in the ER only (n = 1197), survival rate was 25.6 %, with a good/moderate outcome in 19.2 % of cases. A total of 4.8 % in the group with preclinical and ER resuscitation survived, and just 2.7 % had a good or moderate outcome. Multivariate logistic regression analysis revealed the following prognostic factors for survival after traumatic cardiac arrest: prehospital CPR, shock, coagulopathy, thorax drainage, preclinical catecholamines, unconsciousness, and injury severity (Injury Severity Score).

**Conclusions:**

With the knowledge that prehospital resuscitated patients who not reached the hospital could not be included, CPR after severe trauma seems to yield a better outcome than most studies have reported, and appears to be more justified than the current guidelines would imply. Preclinical resuscitation is associated with a higher survival rate and better neurological outcome compared with resuscitation in the ER. If resuscitation in the ER is necessary after a preclinical performed resuscitation the survival rate is marginal, even though 56 % of these patients had a good and moderate outcome. The data we present may support algorithms for resuscitation in the future.

## Background

Patients suffering traumatic cardiorespiratory arrest (TCRA) are generally reported to have a poor outcome [1–12]. A recent systematic review revealed that children appear to have a better chance of survival after resuscitation than adults after suffering out-of-hospital traumatic cardiac arrest, but also that they tend to have a poorer neurological outcome at discharge [13]. Cardiopulmonary resuscitation (CPR) in children after severe trauma seems to yield a better outcome than in adults, and according to a recent study, appears to be more justified than the current guidelines would imply. Resuscitation in the emergency room (ER) was in that study associated with better neurological outcomes compared with resuscitation in a preclinical context or in both the preclinical phase and the ER [14].

The epidemiology of mortality following a polytrauma suggests that as many as 34 % of traumatic deaths occur before hospital arrival [15]. In a German study even 58.7 % of trauma victims died before reaching a hospital [16]. The same authors investigated in another study that 15.2 % (n = 40) of traumatic deaths were classified as preventable [17].

Despite advances in medical treatment and algorithms, only marginal survival rates (0 to 2 %) have been reported for blunt trauma patients who arrive at a trauma centre with no signs of life [4, 8, 12, 18, 19]. Unfortunately, many of these survivors suffer from severe permanent neurological disability [6, 12].

The National Association of Emergency Medical Service (EMS) Physicians/American College of Surgery Committees on Trauma produced guidelines in 2003 on the withholding or termination of resuscitation efforts in out-of-hospital cardiac arrest (OHCA) [19]. Since those guidelines were published, at least two articles have described higher survival rates [1, 20] and one reports a possible deviation from them [20].

In a German study, a tCPR algorithm was introduced, including chest/pericardial decompression, external pelvic stabilization and external bleeding control. The authors showed in their study that prehospital trauma management has the highest potential to improve tCPR and survival [21].

On the other hand, another recent article supported the current guidelines on the withholding or termination of resuscitation of patients in prehospital traumatic cardiopulmonary arrest TCPA [22].

The risk factors associated with different time points of resuscitation (preclinical, ER, or at both time points) were recently published in a huge pediatric population. Here, the population that was resuscitated in the ER revealed the lowest mortality and best outcomes [14]. Not much is known about the prognostic factors for successful resuscitation in the ER of adults suffering TCPA.

The aim of this study was to identify the risk factors and analyze the outcomes of an adult population in the TraumaRegister DGU® (TR-DGU) who were resuscitated in the early post-traumatic phase (prehospital and/or ER).

The TR-DGU collects data and provides a sound basis for even rare events in severely injured trauma patients. The purpose of this study was to analyze the subgroup of patients who required cardiopulmonary resuscitation in the early phase after trauma but were admitted to hospital. Specifically, survival rates and prognostic factors were investigated in this patient group. Beyond survival, the neurological outcome assessed by the Glasgow Outcome Scale (GOS) was considered as well.

## Methods

### TraumaRegister DGU®

The TraumaRegister DGU® (TR-DGU) of the German Trauma Society (Deutsche Gesellschaft für Unfallchirurgie, DGU) was founded in 1993 [23]. The aim of this multi-centre database is an anonymous and standardised documentation of severely injured patients for the purpose of quality control. Data are collected prospectively at four consecutive time periods from the site of the accident until discharge from hospital: (A) prehospital phase, (B) emergency room and initial surgery, (C) intensive care unit and (D) at discharge. This documentation records detailed information on demographics, injury pattern, comorbidities, pre- and in-hospital management, course on the intensive care unit, relevant laboratory findings including data on transfusions, and each individual's outcome. The inclusion criterion is admission to hospital via the emergency room with subsequent intensive care unit (ICU) care. Patients who reach the hospital with vital signs and die before admission to the ICU are included as well.

The infrastructure for documentation, data management, and data analysis is provided by the Academy for Trauma Surgery (AUC - Akademie der Unfallchirurgie GmbH), a company affiliated with the German Trauma Society. The scientific leadership is provided by the Committee on Emergency Medicine, Intensive Care and Trauma Management (Sektion NIS) of the German Trauma Society. The participating hospitals submit their data to a central database via a web-based application. Scientific data analysis is approved according to a peer-review procedure established by the Sektion NIS.

Most of the participating hospitals are located in Germany (90 %), but a rising number of hospitals in other countries have been contributing data as well (these include currently Austria, Belgium, China, Finland, Luxemburg, Slovenia, Switzerland, the Netherlands, and United Arab Emirates). About 35,000 cases from over 600 hospitals are currently being entered into the database per year. Participation in the TR-DGU is voluntary. For certified hospitals associated with the TraumaNetzwerk DGU®, however, participation is obligatory for reasons of quality assurance.

Data anonymity for scientific analyses is guaranteed for both the individual patient and participating hospital. [23–25].

The present study is in line with the publication guidelines of the TraumaRegister DGU® and registered as a TR-DGU project ID 2014-023.

### Patients

We analyzed the 2013 database from 2002 to 2013. Primary admitted adult patients (age ≥16) from Europe with Injury Severity Score (ISS) ≥16 points and available information on CPR (done, or not) both in the prehospital phase and during emergency room (ER) treatment qualified for analysis. Patients transferred in from other hospitals were excluded since prehospital information was missing. Patients declared dead at the accident scene and not transported to a hospital were not recorded in the TR-DGU. The main focus of this survey was the group of patients in cardiac arrest resuscitated in the ER and in the prehospital phase.

A patient’s death can be pronounced outside a hospital and/or clinic setting in Germany, thus the patient is not transported to a medical setting to be declared dead, as happens in some other countries. This requires the diagnosis of at least one definitive sign of death. Patients without return of spontaneous circulation (ROSC) after cardiac arrest were not included in the TR-DGU, according to our inclusion criteria for the registry. This holds true also for patients admitted to the hospital with ongoing CPR but without subsequent ROSC. The registry was established for quality assessment in the acute care hospital and thus did and does not document prehospital deaths.

### Statistical analysis

We performed a descriptive analysis of patients with and without resuscitation in the ER. Then a multivariate logistic regression analysis with resuscitation in the ER as a dependent variable was done to identify independent predictors for survival by calculating odds ratios for each factor. Odds ratios are presented with 95 % confidence intervals (95 % CI). Nagelkerke’s R^2^ was used as an overall goodness-of-fit measure for the model.

The Glasgow Outcome Scale (GOS) was used to classify patients with good or moderate (GOS 4–5) and dead/bad outcome (GOS 1–3) [26]. Furthermore, we carried out a multivariate logistic regression analysis with bad outcome as a dependent variable. Moreover all parameters for the Trauma-Associated Severe Haemorrhage (TASH) score (a score that reliably predicts the probability for mass transfusion after multiple trauma) [27] and coagulopathy (presence of abnormal coagulation parameters upon the patient’s arrival); i.e. prothrombin time test, Quick’s value <70 % and/or platelets <100,000/ml) [28] and acidosis (base excess ≤ -6) were analysed.

Differences between the groups were evaluated by applying chi-squared test for categorical variables and Mann-Whitney *U* test for quantitative and ordinal measures.

Statistical significance was defined as *p <0.05*. Statistical analysis was performed using SPSS Version 20.0 (IBM Corp., Armonk, NY, USA).

## Results

In total 38,499 patients met the inclusion criteria. A prehospital cardiac arrest with subsequent CPR was observed in 1855 patients. These patients had return of spontaneous circulation (ROSC) and were transported to a hospital; the number of cases with attempted but unsuccessful CPR is not documented in the TR-DGU.

The TR-DGU only includes patients who arrived at the hospital. Patients who were dead/who died in the field or patients or without ROSC after cardiac arrest were not included in the TR-DGU, according to our inclusion criteria for the registry.

**Fig. 1:**
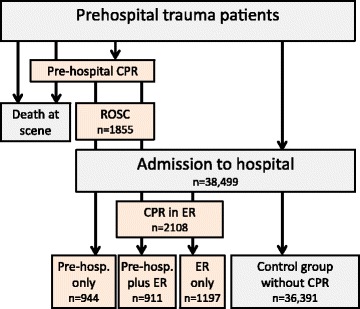
In total 38,499 patients met the inclusion criteria. A prehospital cardiac arrest with subsequent CPR was observed in 1855 patients. These patients had return of spontaneous circulation (ROSC) and were transported to a hospital; the number of cases with attempted but unsuccessful CPR is not documented in the TR-DGU®. Among these cases, 944 patients did not require further cardiac massage in the ER while 911 cases again received CPR in the ER. The total number of patients who required CPR in the ER was 2108 (5.5 % of all admitted patients). Among them were 1197 patients (3.1 %) who were only resuscitated in the ER. The patient subgroups are illustrated in Fig. 1. *CPR* cardiopulmonary resuscitation, *ER* emergency room, *TR-DGU* TraumaRegister DGU®

Among these cases, 944 patients did not require further cardiac massage in the ER while 911 cases again received CPR in the ER. The total number of patients who required CPR in the ER was 2108 (5.5 % of all admitted patients). Among them were 1197 patients (3.1 %) who were only resuscitated in the ER. The patient subgroups are illustrated in Fig. 1.

Epidemiological data are presented in Table 1 covering 2108 patients, who were treated in the ER after only CPR in the ER (n = 1197) and after prehospital and additional CPR in the ER (n = 911). The “Control group” comprises 36,391 patients who were treated in the ER (including 35,477 patients without any CPR and 944 patients after prehospital CPR).

**Table 1:** Epidemiological data are presented covering 2108 patients, who were treated in the ER after only CPR in the ER (n = 1197) and after prehospital and additional CPR in the ER (n = 911)

	Control group	CPR in the ER
	n = 36391	n = 2108
Age (years)	48.1 ± 20.1	49.0 ± 21.0
ISS	27.9 ± 11.0	42.0 ± 18.1
Male/female	27.1 %/72.9 %	29.9 %/70.1 %
Trauma cause		
Car	27.4 %	29.2 %
Motorcycle	14,0 %	13.8 %
Bicycle	8.4 %	7.1 %
Pedestrian	7.8 %	13,0 %
High fall (≥3 m)	19.1 %	19.7 %
Low fall	14.5 %	4.7 %
Others	8.9 %	12.6 %
Traffic accident	41.5 %	35.2 %
Blunt trauma	95.7 %	92.1 %
Penetrating trauma	4.3 %	7.9 %
AIS head ≥3	56.8 %	58.6 %
AIS thorax ≥3	56.2 %	73.6 %
AIS abdomen ≥3	18.4 %	31.8 %
AIS extremities ≥3	33.9 %	46.5 %
Preclinical volume (ml)	1107 ± 864	1601 ± 1155
Time from accident to hospital admission (min)	68.4 ± 29.3	64.0 ± 27.2
Preclinical resuscitation	2.6 %	56.8 %
Preclinical GCS ≤8	31.9 %	70.6 %
Preclinical shock (BP ≤90mmHG)	17.0 %	59.9 %
Preclinical intubation	49.6 %	84.8 %
Preclinical transfusion of catecholamines	9.1 %	53.3 %
Preclinical thoracic drainage	5.4 %	18.0 %
Mild hypotension on admission (BP ≤90mmHG)	13.4 %	63.6 %
Blood transfusion: number of packed red blood cell units	21.9 %	63.6 %
TASH score	6.2 ± 5.0	13.8 ± 6.0
Hemoglobin ER	12.1 ± 2.6	9.1 ± 3.6
Quick’s value ER	79.1 ± 22.9	50.7 ± 28.0
INR ER	1.28 ± 0.65	2.34 ± 2.02
Base excess ER	– 3.0 ± 4.5	– 12.3 ± 9.1
Coagulopathy	29.2 %	70.8 %

The “Control group” comprises 36,391 patients who were treated in the ER (including 35,477 patients without any CPR and 944 patients after prehospital CPR)

*ER* emergency room, *CPR* cardiopulmonary resuscitation, *ISS* Injury Severity Score, *AIS* Abbreviated Injury Severity Score, *GCS* Glasgow Coma Score, *BP* blood pressure, *TASH* Trauma-Associated Severe Haemorrhage, *INR* international normalised ratio

### Mortality

The hospital mortality rate of all resuscitated patient groups was a total of 83.4 % (n = 2545) with patients after preclinical resuscitation alone only amounted to 68.3 % (n = 645), after ER resuscitation only amounted to 74.4 % (n = 891) and patients who required preclinical *and* ER resuscitation had the highest mortality rate with 95.2 % (n = 867).

A total of 1755 patients died after CPR in the ER; there was a mean time to death of 2.3 days (SD 5.8; median 1). Of these 1558 (73.9 % of all, 88.6 % of those who died) died within the first 24 hours after admission. The 350 surviving patients stayed on the ICU for 20.8 days (SD 10.0, median 16.5); their hospital stay was 42.0 days (SD 34.2; median 33.5).

Results of multivariate logistic regression analysis with resuscitation in the ER as a dependent variable (Nagelkerke’s R^2^ = 0.30) are shown in Table 2.

**Table 2:** Results of multivariate logistic regression analysis with resuscitation in the ER as dependent variable (Nagelkerkeʼs R^2^ = 0.3) are shown with n = 29,690 including the 95 % CI

	Koef	SE	*p* value	OR	95 % CI
Preclinical resuscitation	1.747	0.107	**<0.001**	5.74	4.65–7.07
Preclinical catecholamines	0.419	0.091	**<0.001**	1.52	1.27–1.82
Preclinical thorax drainage	0.43	0.102	**<0.001**	1.54	1.26–1.88
Coagulopathy	0.777	0.077	**<0.001**	2.18	1.87–2.53
Preclinical GCS ≤8	0.248	0.081	**0.002**	1.28	1.87–2.53
AIS thorax ≥3	0.23	0.08	**0.004**	1.26	1.08–1.47
AIS abdomen ≥3	0.205	0.078	**0.009**	1.23	1.05–1.43
AIS extremities ≥3	0.236	0.073	**0.001**	1.27	1.10–1.46
Preclinical shock (BP ≤90mmHG)	0.301	0.078	**<0.001**	1.35	1.16–1.57
Mild hypotension on admission (BP ≤90mmHG)	1.250	0.075	**<0.001**	3.49	3.02–4.04
Constant	-5.587	0.100	**<0.001**	0.004	

Statistical significant results are bold. All variables considered in the multivariate models are presented, and all were found to be significant

*ER* emergency room, *SE* standard error, *OR* odds ratio, *CI* confidence interval, *GCS* Glasgow Coma Score, *AIS* Abbreviated Injury Severity Score, *BP* blood pressure, statistic significant results are bold (*p*≤0.005)

The highest risk for mortality in this model was associated with preclinical resuscitation (OR 5.74), followed by shock on admission (OR 3.49), coagulopathy (OR 2.18), preclinical thorax drainage (OR 1.54), preclinical catecholamines (OR 1.52), mild hypotension with preclinical blood pressure ≤90mmHG (OR 1.35), preclinical GCS ≤8 (OR 1.28), relevant injuries of the extremities (OR 1.27), the thorax (OR 1.26), and the abdomen (OR 1.23). The overall injury severity is also associated with in-hospital CPR.

### Neurological outcome

Results of the Glasgow Outcome Scale (GOS) [26] after resuscitation in the preclinical phase and/or in the ER are based on data from 3052 patients. Table 3 displays the Glasgow Outcome Scale (GOS) groups of resuscitated patients.

**Table 3:** Results of the Glasgow Outcome Scale (GOS) [26] after resuscitation in the prehospital phase and/or in the ER based on 38,499 adult trauma patients

Glasgow Outcome Scale	1 Dead	2 Vegetative state	3 Severe disability	4 Moderate disability	5 Good recovery	Good and moderate outcome combined
No resuscitation (n = 35,447)	16.4 % (5816)	2.4 % (865)	12.7 % (4495)	25.4 % (9015)	43.0 % (15,256)	68.5 % (24,271)
Prehospital resuscitation (n = 944)	68.3 % (645)	6.4 % (60)	10.6 % (100)	7.7 (73)	7.0 % (66)	14.7 % (139)
ER resuscitation (n = 1197)	74.4 % (891)	1.3 % (15)	5.1 % (61)	9.7 % (116)	9.5 % (114)	19.2 % (230)
Prehospital + ER resuscitation n = 911)	95.2 % (867)	0.7 % (6)	1.4 % (13)	1.3 % (12)	1.4 % (13)	2.7 % (25)

Pairwise comparison of the three different resuscitation subgroups (*U* test):

ER vs. prehospital: *p* = 0.007

ER vs. prehospital + ER: *p* <0.001

Prehospital vs. prehospital + ER: *p* <0.001

*ER* emergency room

We defined a good or moderate outcome as GOS 5 and 4 and this was analysed in patients without any resuscitation in 68.5. After only preclinical resuscitation the survival rate was with 31.7 % the highest and 14.7 % had a good or moderate outcome (46 % among the survivors). After resuscitation only in the ER, 25.6 % survived in total and 19.2 % had a GOS of 4 or 5 (75.0 % among the survivors). In total 4.8 % in the group with preclinical and ER resuscitation survived and only 2.7 % revealed a good or moderate outcome (56 % among the survivors).

All three analysed subgroups of patients with resuscitation were statistically significant (*p* <0.005; U test) in terms of good and moderate outcome.

Furthermore, we cross-tabulated the prognostic factors for patients with good and moderate outcome (GOS 4 + 5) after resuscitation in the ER, as illustrated in Table 4:

**Table 4:** Prognostic factors for patients with good and moderate outcome (GOS 4 + 5) after resuscitation in the ER using a cross-tabulation

	GOS 4 + 5 (good and moderate outcome) in %	GOS 1–3 (severe disability, vegetative state, dead) in %	Total n =	
ER resuscitation	90.2	52.2	1197	***p*** **< 0.001**
Preclinical + ER resuscitation	9.8	47.8	911	
Penetrating trauma	8.9	7.8	163	*p* = 0.53
Blunt trauma	91.1	92.2	1894	
Female patients	27.2	30.1	628	*p* = 0.38
Male patients	72.8	69.9	1483	
Type of injury				
Car	28.5	29.1	582	*p* = 0.26
Motorcycle	14.6	13,6	275	
Bicycle	6.9	7.1	142	
Pedestrian	9.8	13.4	260	
Fall > 3 m	17.9	20.1	397	
Fall < 3 m	6.9	4.4	94	
Other	15.4	12.2	253	
Severity of injury (AIS)				
AIS head <3	56.0	39.5	881	***p*** **< 0.001**
AIS head ≥ 3	44.0	60.5	1241	
AIS thorax <3	31.5	25.7	560	***p*** **= 0.05**
AIS thorax ≥ 3	68.5	74.3	1562	
AIS abdomen <3	69.6	68.1	1449	*p* = 0.67
AIS abdomen ≥ 3	30.4	31.9	673	
AIS extremities <3	50.6	53.8	1134	*p* = 0.35
AIS extremities ≥ 3	49.4	46.2	988	
Preclinical RR >90 mmHG	73.3	34.9	664	***p*** **< 0.001**
Preclinical RR ≤90 mmHG	26.7	65.1	991	
ER RR >90 mmHG	69.2	31.4	647	***p*** **< 0.001**
ER RR ≤90 mmHG	30.8	68.6	1130	
No blood transfusion	52.5	33.9	700	***p*** **< 0.001**
Blood transfusion	47.5	66.1	1222	
≤10 blood transfusions	79.8	72.8	1418	***p*** **= 0.018**
>10 blood transfusions	20.2	27.2	505	
No coagulopathy	57.8	24.1	471	***p*** **< 0.001**
Coagulopathy	42.2	75.9		
Preclinical GCS >8	70.2	23.8	593	***p*** **< 0.001**
Preclinical GCS ≤8	29.8	76.2	1420	
No acidosis	57.7	20.2	313	***p*** **< 0.001**
Acidosis	42.3	79.8	925	
Age in years	45 ± 20	50 ± 21	2122	***p*** **= 0.005**
ISS	30 ± 12	45 ± 18	2122	***p*** **< 0.001**
Preclinical volume in ml	1302 ± 974	1645 ± 1173	1886	***p*** **< 0.001**
TASH score	9.6 ± 6.3	14.6 ± 5.6	1047	***p*** **< 0.001**
Hemoglobin in the TR	11.2 ± 2.9	8.8 ± 3.5	1777	***p*** **< 0.001**
Quick in the TR	72 ± 25	47 ± 51	1449	***p*** **< 0.001**
INR in the TR	1.40 ± 0.70	2.51 ± 2.14	1482	***p*** **< 0.001**
Base excess	-6.2 ± 7.1	-13.2 ± 9.0	1238	***p*** **< 0.001**

*GOS* Glasgow Outcome Scale, *ER* emergency room, *AIS* Abbreviated Injury Severity Score, *RR* Riva Rocchi, *GCS* Glasgow Coma Score, *ISS* Injury Severity Score, *TASH* Trauma-Associated Severe Haemorrhage, *TR* trauma room, *INR* international normalised ratio, statistic significant results are bold (*p*≤0.005)

Results of multivariate logistic regression analysis with bad outcome (GOS 1–3) as a dependent variable in patients with preclinical resuscitation (Nagelkerke’s R^2^ = 0.28) are shown with n = 841 in Table 5:

**Table 5:** Results of multivariate logistic regression analysis with bad outcome (GOS 1–3) as a dependent variable of the adults after preclinical resuscitation only (Nagelkerke’s R^2^ = 0.28) are shown with n = 841

	Koef	SE	*p* value	OR	95%CI
Preclinical resuscitation	1.131	0.258	***p*** **< 0.001**	3.10	1.9–5.1
ISS (per point)	0.048	0.007	***p*** **< 0.001**	1.05	1.04–1.06
GCS ≤8 preclinical	1.357	0.186	***p*** **< 0.001**	3.886	2.7–5.6
RR ≤90mmHG in the ER	0.909	0.181	***p*** **< 0.001**	2.481	1.74–3.54
Age ≥60 years	1.317	0.199	***p*** **< 0.001**	3.732	2.53–5.52
Blood transfusion	0.534	0.184	***p*** **= 0.004**	1.706	1.189–2.446
Coagulopathy	0.537	0.177	***p*** **= 0.002**	1.711	1.210–2.420
Constant	2.353	0.269	***p*** **<0.001**	0.95	

*GOS* Glasgow Outcome Scale, *SE* standard error, *OR* odds ratio, *CI* confidence interval, *ISS* Injury Severity Score, *GCS* Glasgow Coma Score, *RR* Riva Rocchi, *ER* emergency room, statistic significant results are bold (*p*≤0.005)

## Discussion

Injury is the leading cause of death in industrialised countries of people aged between 1 and 44 years [19, 29, 30], and trauma is the leading cause of death from 1 through 21 years of age [31]. Survival after traumatic out-of-hospital cardiopulmonary arrest in general seems to be rare in the last decades, even with maximum resuscitative efforts. Recently, a study introduced a trauma CPR algorithm and in their collective over 60 % management errors and preventable deaths were found. They concluded that the prehospital trauma management has the highest potential to improve tCPR and survival [21].

In this study we analysed data from the TR-DGU. Our patient cohort consists of victims of severe blunt trauma expressed by a median ISS of 42.2, comparable to other studies in adults on TCRA [12, 32]. Little is known about specific and significant factors during the treatment of polytraumatised patients in terms of different time points of resuscitation (preclinical, in the emergency room or at both times) with specific focus on CPR in the ER. With ample data from the TR-DGU, we have been able to analyse factors and parameters in relation to mortality and neurological outcome as well. Moreover, prognostic factors were analysed.

### Mortality

The 25.6 % survivor rate after resuscitation in the ER we calculated is much higher than the 3.3 % reported in a systematic review containing a meta-analysis reporting a total of 238 survivors out of 5391 patients [13]; our study’s survivor rate is also much higher than that in a retrospective analysis reporting 5.4 % of 1114 patients surviving to hospital discharge after out-of-hospital traumatic cardiopulmonary arrest [33]. The results are similar to the 29 % survivor rate in a prospective observation study from Berlin/Germany [21].

In another study, 5.0 % of the patients underwent resuscitation during trauma room (TR) treatment [14]. In that study, the neurological outcome after only resuscitation in the ER was better than after preclinical resuscitation and after preclinical *and* ER resuscitation. One reason for this finding could be an optimal setting with an interdisciplinary team of specialists vs. a smaller preclinical team with normally only one physician. Another reason might be that cardiac arrest was witnessed in the ER setting in conjunction with resuscitation being initiated immediately, thus keeping the “downtime” minimal.

Moreover the highest rate of survivors was found in the group of preclinical resuscitation with 31.7 %. Unlike in the British and American paramedic systems, the emergency physician in Germany (the *Notarzt*) stabilises the emergency patient at the scene of the accident. The emergency physician in Germany is qualified to declare patients dead at the scene, which is not included in this study. In some countries, paramedic systems cannot declare someone dead without consulting a physician. Thus some trauma victims are transported to the trauma centre just to be declared dead by the trauma physician on call. These differences make comparison with the prehospital phase of other countries’ systems and with international studies therefore difficult.

On-scene triage is done by physicians and is based on their clinical assessment of the patient. These physicians also initiate the early treatment of haemorrhagic shock, provide respiratory assistance, manage pain and sedation, and insert chest tubes if necessary and can also use catecholamines (for sufficient cerebral perfusion, neurogenic shock, resuscitation, etc.). The results from the initial assessment of the patient's condition and those together with the patient’s response to treatment are used by the dispatching physician to find the closest and best-suited facility for the patient. This organisation ensures the availability of resources at the receiving centre and avoids unnecessary secondary transfers from an overcrowded or poorly suited institution (accessed initially because of its proximity to the trauma scene). The cohort demographics including injury patterns are typical for western countries.

Our data fail to support data from earlier investigations by Fulton et al., who emphasised that the location of arrest (on scene, transport or emergency room) and age did not correlate with survival [34]. Our cohort’s survival rates were different after preclinical resuscitation (31.7 %), resuscitation only in the ER (25.6 %), and after preclinical and ER resuscitation (4.8 %). A limitation of the study is that the patients who were dead at the scene were not included in the investigation, because these data are not available.

Our logistic regression model revealed that preclinical resuscitation followed by ISS, preclinical catecholamines, preclinical thorax drainage, coagulopathy, preclinical GCS ≤8 points, AIS thorax, abdomen and extremities ≥3 points, mild hypotension with ≤90 mmHg during the preclinical or ER treatment are strongly predictive for mortality after resuscitation in the ER, in line with findings by Pickens et al., who reported that respiratory efforts and a GCS ≥4 points at the scene have a positive influence on survival, and Durham et al., who reported the same regarding prehospital intubation [20, 35]. Fulton et al. also noted that a loss of neurological function is associated with mortality [34].

The percentage of thoracic trauma in polytrauma patients is generally quite high, ranging from 34 to 70 % [29, 36–39]. Huber-Wagner et al. observed that prehospital chest tube insertion was a strong predictor for survival. Also tension pneumothorax is known as the most common reason for definitive preventable traumatic death [17].

They therefore advised on-scene chest decompression of TCRA patients in case of the decision to initiate emergency cardiac care (ECC) [40].

In the European Resuscitation Council Guidelines for Resuscitation 2015, bilateral chest decompression is advised in the algorithm to address one of the reversible causes for resuscitation [41].

A study by Pickens et al. reported that several survivors met criteria for non-treatment according to proposed clinical guidelines, and that insufficient prehospital treatment was the result [20]. Katz et al., for example, analysed a 25 % rate of misplaced tracheal tubes by paramedics (27/108) [42]. Furthermore, Cera et al. found that the time of intubation (on-scene versus in-hospital) did not influence survival [1].

Normally, patients in a clinical setting should never be deprived of sufficient oxygen for a relevant period of time, unlike what can happen in a preclinical setting, where patients probably suffer several minutes without oxygenation before the medical staff reaches the scene. The fact that a patient requires resuscitation in a preclinical and ER setting seems to reflect the trauma’s severity and according to the literature often appears to be associated with a poor outcome.

In our investigation among 31.7 % survivors in the group of preclinical resuscitation 46 % had a good or moderate neurological outcome; after resuscitation in the ER 25.6 % survived, with 19.2 % having a GOS 4 or 5.

GOS has been documented in the TR-DGU since 2002, thus there are no GOS records for patients from 1993 to 2001. Our GOS calculation is therefore based on 38499 patients.

Reliable data describing outcomes in detail and including neurological function are rare in the literature. A recently published literature review covering 1980 to 2011 analysed the outcomes of children treated for out-of-hospital traumatic cardiopulmonary arrest who reached the clinic alive: 29 patients (57 %) were severely disabled; 3 children (6 %) moderately disabled, and 19 patients (37 %) had a normal outcome [43]. Margolin et al provide information on the GOS in six of 13 survivors (46.2 %) out of 67 patients (79.1 % blunt trauma) who had at least a satisfactory outcome as expressed by a GOS of ≥4 [32]. Other studies describe patients’ outcome data in terms of their being “neurologically intact” after TCRA: Fialka et al. observed 100 % of patients surviving with no neurological deficit (4/4, n = 38) [44], Fulton et al. 83.3 % of survivors being neurologically intact (5/6, n = 245) [34], Powell et al. 81.0 % of survivors (21/26, n = 959) [11], Stratton et al. 66.6 % (3/9, n = 79) [8], Battistella et al. 56.3 % (9/16, n = 604) [6], Branney et al. 3.9 % (34/42, n = 950) [45], and Martin et al. no neurologically intact patients (0/1, n = 110) [12].

Yanagawa et al. detected no significant differences between out-of-hospital TCRA patients with or without head injury except for the fact that the blunt traumatic patients with a head injury more often displayed a return to spontaneous circulation (ROSC). However, they observed no head-injury effect on the outcome [3]. In our investigation, a preclinical GCS ≤8 had a significant odds ratio of 2.18 in the multivariate logistic regression analysis with resuscitation in the ER as a dependent variable. Moreover, an AIS head ≥3 was a significant prognostic factor in our cross-tabulation, with a GOS 4 and 5 and resuscitation in the ER and preclinical GCS ≤8 showing a significant odds ratio of 3.886 in multivariate logistic regression analysis with GOS 1–3 as a dependent variable.

Our multivariate logistic regression revealed that injury severity, the preclinical use of catecholamines and preclinical thorax drainage, coagulopathy, a preclinical GCS ≤8, severe injuries to the thorax, abdomen and extremities, and preclinical shock in the ER are strongly predictive of resuscitation in the ER.

Sorted according to a bad prognosis with GOS 1–3, these factors were preclinical resuscitation, ISS, preclinical GCS ≤8, mild hypotension in the ER with Riva Rocchi (RR) ≤90mmHG, blood transfusion and catecholamines, and age ≥60 years.

Base deficit is a rapidly and widely available serum laboratory marker of systemic acidosis that increases with hypoxemia and/or shock. In the trauma setting, the base deficit correlates with the blood-transfusion requirement, risks of multiorgan failure and mortality in adult patients, including those with traumatic brain injury [31–33].

These findings are in line with the sparse data in the literature reporting that blood pressure = 0 [34] or low blood pressure in the field were prognostic factors [35].

### Limitations

Our study has several limitations. Patients declared dead on-scene without transportation to a hospital were not recorded in the database. Moreover, only patients surviving CPR and transportation to the hospital could be included. That means that our database does not include those patients who required prehospital CPR but did not achieve ROSC. This is because the TraumaRegister DGU® was established as a tool for quality assessment in the acute care hospital. Patients who died before admission were thus not relevant to such an assessment. Furthermore, Germany maintains no documentation system that covers all trauma cases, including all prehospital deaths. Our survival rates thus do not refer to all cases with cardiac arrest after trauma but only to those cases who were admitted to the acute care hospital alive, i.e., with ROSC. This limitation and bias must be kept in mind when interpreting the surprisingly good survival rates.

This and other exclusion criteria (ISS <16 and secondarily transferred patients) were excluded. This could have biased the results.

Due to the fact that ECG data are not documented in the TR-DGU®, no information on cardiac rhythm was available. Furthermore, we had no specific information on the exact duration of chest compression in the pre- and/or in-hospital phase. According to the literature, resuscitation lasting more than 20 minutes seems to be associated with poor neurological outcome [33]. We could only analyse patients who underwent closed-chest compression on-scene or during transport and/or during treatment in the ED. The main limitation is our patient cohort’s preselection. The TR-DGU® only includes patients who have been transported to a hospital after chest compression after TCRA. This might be one reason for the relatively high survival rates compared to other studies. The group investigated in our study represents patients who had been resuscitated because of a subjective assessment by the on-scene emergency physician who perceived a relatively certain chance of survival for those patients.

## Conclusions

According to our data, CPR in patients after severe trauma seems to yield a better outcome than most studies have reported, and it appears to be more justified than the latest guidelines would imply. The results potentially alter the attitude towards the usefulness of intensive resuscitation in patients suffering trauma.

Prehospital resuscitation is associated with higher survival rate compared with resuscitation in the ER. Overall, good and moderate neurological outcomes were highest in the group of only ER resuscitation and among the survivors in the prehospital resuscitation group.

Resuscitation in both the preclinical phase and the ER had fatal mortality rate, even though among the survivors the rate of patients with a GOS 4 and 5 was quite high. We hope that our findings will help future patients by facilitating the drafting of guidelines and algorithms for the resuscitation of polytraumatised patients.

## Key messages

- Patients’ outcomes after severe trauma seems to be better than those reported in most previous studies.
- Resuscitation in the ER is associated with better neurological outcomes compared with resuscitation in a preclinical context or in both the preclinical phase and the ER.
- Among the survivors, patients who survived preclinical resuscitation had the highest rate of good or moderate outcome
- These data might support algorithms and guidelines in the future dealing with the resuscitation of polytraumatised patients.

## Abbreviations

AIS: Abbreviated Injury Severity Score
AUC: AUC - Academy for Trauma Surgery (AUC - Akademie der Unfallchirurgie GmbH)
CI: Confidence interval
CPR: Cardiopulmonary resuscitation
DGU: Deutsche Gesellschaft für Unfallchirurgie
ECC: Emergency cardiac care
EMS: Emergency medical services
ER: Emergency room
GCS: Glasgow Coma Scale
GOS: Glasgow Outcome Scale
ICU: Intensive care unit
ISS: Injury Severity Score
NISS: New Injury Severity Score
OHCA: Out-of-hospital cardiac arrest
ROSC: Return of spontaneous circulation
RR: Riva Rocchi
Sektion NIS: Notfall-, Intensivmedizin und Schwerverletztenversorgung
TCPA: Traumatic cardiopulmonary arrest
TCRA: Traumatic cardiorespiratory arrest
TR-DGU: TraumaRegister DGU®
